# Debates in allergy medicine: food intolerance does not exist

**DOI:** 10.1186/s40413-015-0088-6

**Published:** 2015-12-14

**Authors:** Sten Dreborg

**Affiliations:** Women’s and Children’s Health, University of Uppsala, Uppsala, Sweden

**Keywords:** Nomenclature, Immunologically mediated hypersensitivity, Allergy, Non-immunologic hypersensitivity, Non-allergic hypersensitivity, Intolerance, IgE sensitization, Non-IgE-mediated allergy, IgE-mediated allergy, Cell mediated allergy

## Abstract

**Background:**

The term “intolerance” is not mentioned in the World Allergy Organization (WAO) document on allergy nomenclature. “Intolerance” has been used to describe some non-immunological diseases. However, pediatric gastroenterologists mix allergy and intolerance, e.g. by using the term “cow’s milk protein allergy/intolerance (CMPA/I)”, lumping together all types of mechanisms for not tolerating cow’s milk. The basis for this mix is the fact that double-blind oral food challenges are time-consuming and expensive. Therefore, cow’s milk exclusion and reintroduction is proposed to be used in primary care for the diagnosis of CMPA in children with common gastrointestinal (GI) problems such as colic and constipation. This may lead to a widespread use of hypoallergenic formulas in children without proven CMPA. In lay language, *intolerance* describes “not tolerating”.

**Objective:**

To discuss the reasons why the term “intolerance” should not be used in the area of allergy.

**Results:**

Presently, *intolerance* is not part of the allergy nomenclature. It is used by lay persons to describe “not tolerating”. Pediatricians use *intolerance* to describe non-immunological hypersensitivity such as lactose intolerance which is acceptable. However, using the mixed term CMPA/I describing a variety of gastrointestinal symptoms in children, should be avoided. The WAO Nomenclature does not clearly distinguish between non-IgE-mediated allergy and non-allergic hypersensitivity.

**Conclusion:**

The term “intolerance” should not be used within the area of allergy. *Intolerance* should be better defined and the term restricted to some non-immunological/non-allergic diseases and not mixed with allergy, e.g. by using the term CMPA/I. A revision of the WAO nomenclature is proposed.

## Background

**Fig. 1:**
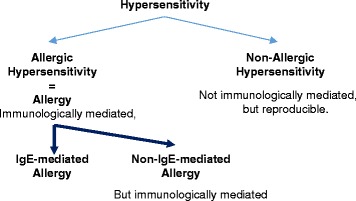
The principle of the EAACI/WAO nomenclature. (Original work of the author)

When asked to write the “con” paper for the Journal entitled “Food intolerance does not exist”, I felt it was a rather simple task. According to the World Allergy Organization (WAO) agreement on allergen nomenclature [1], intolerance does not exist. The nomenclature agreement is based on mechanisms. Hypersensitivity is either non-immunological or immunological, i.e. allergy. Allergy can be either IgE-mediated or due to other mechanisms. The WAO document does not go into detail and leaves out subdivision of non-allergic hypersensitivity. The nomenclature agreed upon seems straightforward and non-controversial. The basic principle is illustrated in Fig. 1.

### Conclusion

Intolerance does not exist.

However, despite the fact that allergists do not use intolerance to describe allergy or allergic diseases, the word intolerance is used by gastroenterologists and lay persons, which should be discussed in this context.

#### Cow’s milk protein allergy/intolerance, CMPA/I

In 2012 Vandenplas [2] stated: “The old term ‘intolerance’ gives rise to confusion. To avoid this, ‘intolerance’ is proposed to be restricted to the incapacity to fully digest carbohydrates, mainly disaccharides, of which lactose is the most important one.” If restricted to lactose, fructose, sucrose etc., the term “intolerance” can be acceptable, since it does not describe allergic or immunologic hypersensitivity conditions, and implies there is no immunological, i.e. allergic, mechanism involved.

Furthermore, Vandenplas states: “A cow’s milk challenge is the gold standard for the diagnosis of CMPA but does not provide proof that the immune system is involved” [3, 4]. That is correct. However, for the diagnosis of an allergic condition an immunological mechanism must cause the reaction, either IgE-mediated or by other less well-known mechanisms that cannot be diagnosed by any simple diagnostic tool.

The next step in the argumentation has been to lump together cow’s milk protein allergy (CMPA) and cow’s milk protein intolerance (CMPI) to CMPA/I, i.e. to mix allergic and non-allergic reactions. During the “Fifth European pediatric motility meeting”, Vandenplas et al. [4] wrote under “Gastrointestinal Manifestation of Cow’s Milk Protein Allergy or Intolerance and Gastrointestinal Motility”: “The overlap between gastrointestinal manifestations of cow’s milk protein allergy or intolerance (CMPA/I) and frequent (functional) gastrointestinal complaints such as gastro-esophageal reflux (disease) (GER(D)) and constipation are a topic of debate since many years. The debate is the logic consequence of the fact that objective diagnostic criteria for each of the entities are missing. Since not one sign or symptom is specific for CMPA or CMPI, and since the same is valid for GER(D) and constipation, and since all conditions are relatively frequent, it is a given fact that some of the allergic infants will present with reflux and/or constipation and that some of the infants with reflux or constipation will have CMPA/I.” This is not acceptable. Allergy may not be used unless an immunologic mechanism is proven or highly probable. In cases where there is no immunological mechanism, the term “allergy” may not be used. The goal must be to differ, as far as possible, between immunological, i.e. allergic and non-immunological mechanisms.

Vandenplas et al. continue: “If the reintroduction of CMP causes relapse of symptoms, the diagnosis seems established, since a challenge-test is considered as the golden standard diagnostic test. Although false positive diagnostic testing (specific IgE, skin prick test, patch test) does occur, the diagnosis of CMPA is ‘likely’ if patients present with suggestive symptoms and (one of) these tests show positive results. But, sensitization with CMP can also lead to non-IgE dependent reactions. Some symptoms are more frequently linked to non-IgE mediated allergy”. . . . “In daily routine, there is no diagnostic testing for non-IgE-mediated allergic reactions”. This statement allows for the possibility of unrestricted use of CM avoidance, i.e. use of hypoallergenic formulas, in infants with common non-specific complaints. This is not evidence based.

The conclusions by Vandenplas seem to be: Oral provocations are difficult to perform in general practice and do not indicate mechanism, i.e. IgE-mediated allergy, non-IgE-mediated allergy or non-allergic hypersensitivity. Therefore, these diagnoses are lumped together under CMPA/I.

Allergen specific IgE tests indicate sensitization not allergic disease. To prove CMPA, a history of immediate reaction to CMP should be supported by an oral provocation, unless clear severe allergic symptoms appeared in close connection with food intake. Then, with a suggestive clinical history, atopic sensitization, and a supervised oral provocation test, the infant has been proven to have an atopic/IgE mediated CMPA. With any of the common symptoms, but without IgE sensitization, the infant does not have IgE-mediated allergy to CMP. Then two possibilities remain; non-IgE-mediated allergic CMPA and non-allergic CMP hypersensitivity. To differentiate between these conditions is often not easy. There are no simple diagnostic methods diagnosing non-IgE-mediated allergy. According to Vandenplas et al. [4] symptoms common in infants (Table 1) may indicate CMPA/I. Since performing oral challenges is claimed to be difficult, Vandenplas introduced a scoring system [5], Table 2, as a basis for an algorithm [6] to be used in general practice or by practicing pediatricians based on the degree of common gastrointestinal symptoms, atopic eczema and some respiratory symptoms.

**Table 1:** Most frequent symptoms of mild to moderate CMPA according to [4]

Therapeutic area	Symptoms
Gastrointestinal	
	Frequent regurgitation
	Vomiting
	Diarrhea
	Constipation^a^
	Blood in stool without failure to thrive
Dermatological	
	Atopic dermatitis
	Swelling of lips or eye lids
	Urticaria unrelated to acute infections, drug intake, or other causes
Respiratory	
	Runny nose
	Recurrent otitis media
	Chronic cough
	Broncho-constriction unrelated to infection
General	
	Persistent distress
	Colic (≥3 h/day wailing/irritable) over a period of >3 weeks

Infants with CMPA in general show one or more of the listed symptoms

*CMPA* cow’s milk protein allergy

^a^Compare type of stool in Table 2, my remark

**Table 2:** Symptom-based score according to Vandenplas et al. [5]

Symptom	Score			
Crying	0–6	0: 1 h/day		
		1: 1–1.5 h/day		
		2: 1.5–2 h/day		
		3: 2–3 h/day		
		4: 3–4 h/day		
		5: 4–5 h/day		
		6: >5 h/day		
Regurgitation	0–6	0: 0–2 episodes/day		
		1: ≥3 to ≤5 of small volume		
		2: >5 episodes of >1 coffee spoon		
		3: >5 episodes of ± half of the feedings in < half of the feedings		
		4: continuous regurgitations of small volumes > 30 min after each feeding		
		5: regurgitation of half to complete volume of a feeding in at least half of the feedings		
		6: regurgitation of the complete volume after each feeding		
Stools (according to Bristol scale)	0–6	4: type 1 and 2 (hard stools)^a^		
		0: type 3 and 4 (normal stools)		
		2: type 5 (soft stool)		
		4: type 6 (liquid stool, if unrelated to infection)		
		6: type 7 (watery stools)		
Dermatological symptoms	0–6	Atopic eczema		
		Head–neck–trunk		Arms–hands–legs–feet
		Absent	0	0
		Mild	1	1
		Moderate	2	2
		Severe	3	3
	0–6	Urticaria (no: 0/yes: 6)		
Respiratory symptoms	0–3	0: no respiratory symptoms		
		1: slight symptom		
		2: mild symptoms		
		3: severe symptoms		

^a^Compare constipation in Table 2, my remark

(Permission received from John Wiley & Sons, Ltd.)

The problems are

- Symptoms in the scoring system [5], possibly indicating CMPA/CMPI, include symptoms exhibited by the majority of infants. In the majority of cases, these symptoms are not based on an immunologic mechanism, Table 1 [4]. However, since proper Double Blind Placebo Controlled Food Challenge (DBPCFC) in infants with colic or constipation have not been published so far, the scientific proof for the presence of any CMPA/I in infants with such symptoms is lacking.
- Non-IgE-mediated allergy and non-allergic hypersensitivity are lumped together.
- Reintroduction [4] of cow’s milk at home without supervision and retrospective evaluation by a general practitioner or general pediatrician is considered equal to oral provocation supervised by a specialized team, naming it oral provocation, the gold standard.

According to the argumentation by Vandenplas et al., any gastro-intestinal symptom may be caused by non-IgE-mediated CMPA/“intolerance” (CMPA/I), e.g. colic and constipation, Table 1. This type of argumentation is the impetus for the widespread use of non-specific criteria for the diagnosis of “CMPA/I” in primary care. In a web-based survey among practicing pediatricians in Europe [7], “the prevalence of infants presenting with CMA was 47 % as perceived by general pediatricians. Eczema, vomiting, diarrhea, rashes/hives, blood in stools and a symptom duration of more than 1 week were features associated with CMA. Only 21 % of the doctors performed diagnostic allergy tests including cow’s milk-specific serum IgE or skin prick test. Sixty seven percent of the responding general pediatricians indicated clinical signs alone or an empirical trial of a replacement formula were sufficient for diagnosis” [7]. A hypoallergenic CM formula was the most commonly used elimination diet. However, 20 % of practicing pediatricians used amino acid based formulas in these children. This non-evidence-based clinical decision may generate anxiety among parents, unnecessary elimination diets, disabling diagnoses, and un-necessary expenses to parents and even the society, in some countries.

Fortunately, the European Society of Pediatric Gastroenterology Hepatology and Nutrition (ESPGHAN) position paper [8] on the management of CMPA does not use the confusing term CMPA/I.

#### The present nomenclature

When preparing this article, I read the nomenclature papers by EAACI [9] and WAO [1] once again, concentrating on gastrointestinal problems, which is most often part of the “intolerance” discussion. I did not find any “intolerance” in these documents.

However, in the EAACI position paper [9], it is proposed that “an adverse reaction to food should be called *food hypersensitivity* …. When immunologic mechanisms have been demonstrated, the appropriate term is *food allergy*, and, if the role of IgE is highlighted, the term is *IgE-mediated food allergy*”. So far, correct. However, the authors continue, “All other reactions, previously sometimes referred to as ‘food intolerance’ , should be referred to as *non-allergic food hypersensitivity*”. Thus, the group of non-IgE-mediated mechanisms is not mentioned in that text or as being part of food intolerance.

**Fig. 2:**
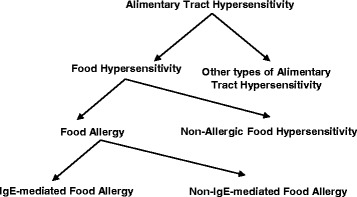
The principle of the EAACI/WAO nomenclature adopted to food allergy. (Original work of the author)

In the WAO document [1], the definitions are clear in the general introduction (Fig. 2). However, under “Food allergy” it is stated: “The appropriate term is *food allergy* when immunologic mechanisms have been demonstrated.… If IgE is involved in the reaction, the term IgE-mediated food allergy is appropriate. All other reactions should be referred to as non-allergic food hypersensitivity”. There is a gap in not mentioning non-IgE-mediated allergic mechanisms.

The matter of these two exclusions probably confuses some readers and opens up the possibility for using different symptoms to diagnose CMPA that includes non-IgE-mediated CMPA, and to add “I”, i.e. using the term CMPA/I. The wording of the WAO nomenclature position paper should be changed to indicate that food allergy caused by non-IgE-mediated mechanisms exists.

#### Future use of intolerance

“Intolerance to” is often used by lay persons to describe “not tolerating” or “getting symptoms from contact with” without bothering with the mechanism causing the “intolerance”, thus rather similar to the EAACI/WAO term “hypersensitivity”. Since intolerance is an accepted term, but not indicating allergy, maybe *intolerance* could be used as a substitute for *non-allergic/non-immunological diseases*, which is an awkward. *Intolerance* could be introduced as a shorter term for *non-immunological hypersensitivity* similar to using *allergy* instead of *immunologically mediated hyperreactivity*. This would also have a positive effect: in the future *intolerance* will be opposite to *allergy*, i.e. it will indicate absence of any type of allergy, or immunological mechanism.

In addition, I believe it would be of value to discuss defining different non-IgE-mediated and non-allergic (intolerant?) diseases in a future nomenclature position paper by WAO. Finally, since all mechanisms involved are not fully understood, an easily understood common name for non-IgE-mediated allergy/non-IgE-mediated immunological hypersensitivity should be introduced.

In the WAO position paper, non-atopic eczema is mentioned, but *non-atopic* is not used for food allergies. Thus, *non-IgE-mediated hypersensitivity/allergy* is proposed be named, “non-atopic food allergy”, which is the opposite of *IgE-mediated food hypersensitivity* or *atopic food allergy*.

**Fig. 3:**
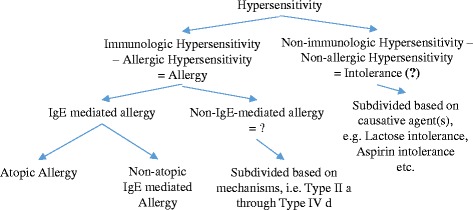
Illustration of proposed minor changes of the food hypersensitivity nomenclature, using “intolerance” as short for “non-allergic hypersensitivity”/“non-immunological hypersensitivity”. (Original work of the author)

Dividing “non-IgE-mediated food allergy” based on the revised Gell and Coombs nomenclature [10], as proposed by Uzzaman and Cho, should be discussed [11]. The proposed basic principle is shown in Fig. 3, including a proposal for sub-division of “non-IgE-mediated allergy” adding the present sub-groups of Type II and IV allergy.

#### Intolerance in media

The use of “intolerance” in lay media is often confusing. As an example, I refer to Wikipedia, Medical/biological intolerance (Table 3) [12]. The introduction, “*Intolerance*, or *hypersensitivity,* includes but is not limited to allergy” is highly confusing. This introduction and several of the intolerances mentioned are certainly not acceptable (Table 4).

**Table 3:** Medical/biological intolerance according to Wikipedia [12]^a^

Intolerance, or hypersensitivity, includes but is not limited to allergy	
• Cold intolerance • Drug intolerance • Exercise intolerance • Fructose malabsorption • Heat intolerance • Hereditary fructose intolerance • Lactose intolerance • Lysinuric protein intolerance	• Multiple chemical sensitivity • Orthostatic intolerance (?) • Perfume intolerance • Salicylate intolerance, also known as aspirin intolerance • Sucrose intolerance • Food intolerance • Gluten sensitivity • Milk soy protein intolerance

^**a**^Downloaded on June 1 2015

**Table 4:** The types of allergic mechanisms as described by Gell and Coombs [10], revision as proposed by Uzzaman and Cho [11]

No	Type	Mechanism	Disease
I	Immediate type allergy	IgE	Anaphylaxis Atopic asthma, rhino-conjunctivitis immediate type urticaria etc.
II a	Cytotoxic or IgG/IgM mediated	IgG/IgM	Auto-immune diseases
II b	Antibody-mediated cell stimulating	Antibodies cell stimulation	Diffuse goitre Basedow’s/Grave’s disease “Autoimmune” chronic idiopathic urticaria
III	Immune complex mediated -	IgG/IgM - complement	Lupus erythematosus Epidermolysis bullosa
IV a	Macrophage activation	CD4(+)Th1 lymphocyte mediated with activation of macrophages	Granulomatous diseases Type I diabetes mellitus
IV b	CD4(+)Th2 eosinophilic reaction	CD4(+)Th2 lymphocytes and eosinophils	Chronic asthma and chronic rhinitis
IV c	Cytotoxic CD8(+) T lymphocyte incused apoptosis	Cytotoxic CD8(+) T lymphocytes Perforin-granzme B apoptosis	Stevens-Johnson syndrome Toxic epidermal necrolysis
IV d	T-lymphocyte-driven neutrophilic inflammation	T-lymphocytes Neutrophilic inflammation	Pustular psoriasis Acute generalized exanthematous pustulosis

Furthermore, Wiktionary informs the following: “Intolerance: sensitivity to a food or drug; allergy. (medicine): food intolerance: the state of being intolerant; extreme sensitivity to a food or drug; allergy” [13].

#### Future nomenclature

I would propose that *non-IgE-mediated allergy* be subdivided according to Gel and Coombs; and allergic diseases, and diseases or symptoms with an obvious immunological background, but not yet with a fully understood mechanism [14], are mentioned under this heading. Furthermore, the often-used word “tolerance” with different contextual meanings, should be better defined unanimously, and *sensitization* vs. *clinical allergy* should be better defined.

To influence the use of “intolerance”, it is essential that the WAO extends its nomenclature document with a more detailed description of non-immunological diseases (intolerance?), by collaborating with competent adult and pediatric sister societies. Then, it is essential that the nomenclature be spread to relevant journals, societies and lay organizations.

## Conclusions

- Intolerance does not exist within the area of allergy and should not be part of the allergy/immunological hypersensitivity nomenclature.
- The mixed term “cow’s milk protein allergy/intolerance” (CMPA/I) should be actively counteracted in collaboration with related societies.
- *Intolerance* is proposed to indicate gastrointestinal lack of enzyme-causing GI symptoms (lactose, fructose, sucrose intolerance).
- *Intolerance* or any other easily recognized term should be added to *non-allergic/ non-immunologic hypersensitivity* which is too awkward to be generally accepted. This action will clearly separate *non-IgE-mediated hypersensitivity/allergy* from *non-immunologic hypersensitivity* (/intolerance).
- *Non-IgE-mediated hypersensitivity/allergy* should be given a shortened name.

Other terms often used in discussions on allergy, such as “tolerance”, “sensitization” vs. “clinical allergy” and “de-sensitization”, should be better defined.
